# Dietary Diversity and Nutritional Status of Preschool Children in North West Province, South Africa: A Cross Sectional Study

**DOI:** 10.3390/children7100174

**Published:** 2020-10-09

**Authors:** Perpetua Modjadji, Dineo Molokwane, Patricia Ogechi Ukegbu

**Affiliations:** 1 School of Health Care Sciences, Department of Public Health, Sefako Makgatho Health Sciences University, 1 Molotlegi Street, Ga-Rankuwa 0208, South Africa; dineomolokwane78@gmail.com; 2 Department of Human Nutrition and Dietetics, Michael Okpara University of Agriculture, Umudike PMB 7267, Abia State, Nigeria; adanna2025@yahoo.com

**Keywords:** dietary diversity, nutritional status, preschool children, peri-urban, South Africa

## Abstract

Preschool children consume diets inadequate to meet their macro and micronutrient requirements, which ultimately affect their nutritional status due to lack of dietary diversity. A cross sectional study was conducted to investigate the association between dietary diversity scores (DDS) and the nutritional status of 379 preschool children in North West Province of South Africa. A 24 h qualitative recall by mothers of their children’s food consumption was used to calculate DDS based on 12-foods groups following Food and Agriculture Organization protocols. DDS was calculated by counting each of 12-food groups and classified as low (≤4), medium (5–8) and high (9–12). The weight and height of children were measured and height-for-age (HAZ), weight-for-age (WAZ) and BMI-for-age (BAZ) z-scores were calculated based on 2006 WHO standards. Stunting, underweight and thinness were defined as HAZ, WAZ and BAZ < −2SD, respectively. Linear and logistic regression analyses were used to assess the association between DDS and the nutritional indicators. Mean age for children was 4 ± 0.7 years, and the prevalence of stunting (29%), underweight (13%) and thinness (6%) was observed. Mean DDS was 4.39 ± 1.55 out of 12-food groups, with a prevalence of 61% and 39% for low and medium DDS, respectively. Cereals (100%) accounted for the main food group consumed, while fish and other seafood (17%) were the least consumed. Consumption of a diversified diet was associated with lower odds of being stunted [AOR = 0.25, 95%CI: 0.10 to 0.92] among the four-year olds, while in the unadjusted model, 5-year-olds had lower odds of being underweight [OR = −0.32, 95%CI: −0.57 to 0.07]. The findings of this study reinforce the importance of continued nutrition education of mothers, caregivers and preschool staff on the need to ensure consumption of diverse food sources in order to improve the nutritional status of children. Further studies are recommended on the association of DDS with the nutritional status, and factors associated with low dietary diversity among preschool children.

## 1. Introduction

Dietary diversity, defined as the total number of food groups consumed over a reference period, has gained prominence as a valid and reliable indicator of dietary adequacy among children [1]. The diversity of foods provided to young children, particularly meat, poultry, fish, eggs, fruits and vegetables, is recommended to improve micronutrient intakes [2]. Nonetheless, lack of dietary diversity is a severe problem among poor populations from the developing world, especially in Africa [1,3]. Most of the diets consist of monotonous starchy staples, and often include little or no animal products and few fresh fruits and vegetables [4]. Dependency on plant-based staples such as maize meal, in addition to low-cost fats and sugar, are barriers to optimal feeding [5]. The World Health Organization has recommended a minimum dietary diversity of at least four food groups out of seven in order to maintain proper child growth and development [6], but many children cannot meet this criterion [7].

The 2015 Millennium Development Goal has reported that one third of all children who are undernourished in the world live in sub-Saharan Africa (SSA) [8]. In these countries, the adequacy and diversity of diet in most households are affected by poverty, insufficient knowledge, social circumstances, cultural beliefs and practices of caregivers [5,9]. In particular, the relationship between dietary diversity and the nutritional status of preschool children is well-established [10,11]. Inadequate diversified diet characterized by the deficiency of macronutrients and micronutrients results in poor nutritional status indicated by stunting (low height-for-age), underweight (low weight-for-age) and wasting/thinness (low body mass index-for-age), as well as ill health [12]. In SSA, most children under five years are stunted (39%), followed by underweight (25%), and wasting (10%) [13]. These prevalence are suggestive of the nutrient inadequacies of the diet [14]. The challenge to connect poor growth and specific nutrient deficiencies has been acknowledged, due to the need for multiple nutrients required for growth and development. Henceforth, dietary diversity has been proposed as a candidate indicator of food security and a predictor of nutritional status [14].

In South Africa, the National Consumption Survey has reported a low dietary diversity, low energy and inadequacy of certain essential micronutrients among children [15]. Although, to some extent, the introduction of mandatory fortification of commercial maize meal in South Africa has reduced deficiencies of some nutrients among adults [16,17], poor dietary diversity is still a concern in the country [17]. The odds of children not benefiting from food fortification in South Africa have been reported, mainly because of the small amounts they consume [18]. One out of two children in the country were reported to have an energy intake of less than two-thirds of their energy needs [15]. Most of the children consume a diet with poor nutrient density to meet their macronutrient and micronutrient requirements in the country [15,19]. Furthermore, the prevalence of stunting, underweight and wasting have been estimated at 27%, 5.9% and 2.5%, respectively, among children under five in South Africa [20].

Most children spend a large part of the day at childcare facilities (CCFs) worldwide [21]. CCFs include preschools, which are a learning space environment offering early childhood education to children before they begin compulsory education at primary schools, and are regarded as early childhood development (ECD) services [22,23]. ECD services provide education and care to children, including nutritional care, in the temporary absence of their parents or adult caregivers [21,23]. However, food menus offered to children in these facilities are nutritionally inadequate [21], which ultimately affects their nutritional status due to lack of dietary diversity. ECD programmes are regarded as a strategy to alleviate poverty, according to the Social Development Ministry in South Africa [23]. The South African government subsidises the ECD programmes with amounts of ZAR9 to ZAR12 per child, mainly to be used towards the full stay of the child and food availability [23]. A corporation of parents and preschool owners to provide nutrient-rich foods to ensure optimal nutrition for developing children has been documented [24].

Data on the association between dietary diversity and the nutritional status of preschool children are limited in South Africa [25,26]. Most studies in the country have focused on other aspects of child nutrition, like micronutrient content [27,28], feeding practices [29,30] and malnutrition and its determinants [31,32], but less on the relationship between dietary diversity and the nutritional status. In view of this, the main objective of the study was to quantify dietary diversity scores and the nutritional status indicators, and determine their association among children attending preschools in North West Province of South Africa. Understanding the influence of dietary diversity on the nutritional status of children provides useful information to enhance interventions that focus on improving the quality of diets. Findings from this study will therefore contribute significantly to public health programmes aimed at ending all forms of malnutrition and hunger by 2030 [33].

## 2. Methods

### 2.1. Study Design

This was a cross-sectional study conducted to investigate the association between dietary diversity and the nutritional status of preschool children in North West Province, South Africa. The study was conducted from June to December 2019.

### 2.2. Study Setting

The study was conducted in Dr. Kenneth Kaunda District, situated in the North West Province of South Africa. The District is one of the four districts of the Province and is divided into three local municipalities; JB Marks (town; Potchefstroom), Maquassi Hills (town; Wolmaransstad) and Matlosana City (town; Klerksdorp). Dr. Kenneth Kaunda District has a population 742,821 people, and the inhabitants mainly speak Setswana. This District was selected as the area of interest for this study, while Klerksdorp and Wolmaransstad as peri-urban areas and were considered due to the scarcity of nutritional studies among children in these areas compared to Potchefstroom. Dr. Kenneth Kaunda District has 116 preschools. We used preschool settings, because most children who have not reached primary school age are under care in these facilities, which parents are reliant on. Furthermore, concerns regarding the food menus at these facilities to achieve adequate dietary specifications have been raised [21].

### 2.3. Study Participants

The study included children aged three to five years attending preschools in Dr. Kenneth Kaunda District, who had no physical disabilities that would have affected their stature, whose mothers gave consent and were available to participate in the study.

### 2.4. Sample Size and Sampling Procedure

The total enrolment number of children aged between three and five years in the preschools of Klerksdorp and Wolmaransstad municipalities was estimated at 3600 through personal communication with the heads of preschools, with enrolments ranging from a total number of 40–45 in the unfunded preschools to enrolments of 80–126 in the largest (funded) preschools. No electronic database on the enrolment numbers was available at the time of the study. Rao software was used to calculate a sample size, taking into consideration the population size of 3600 children, a 5% margin of error, and 95% confidence level. A minimum representative sample of 348 was calculated and buffered with 10% to make up for non-responses, and a sample size of 415 was obtained. Multi-stage sampling was used to select facilities and participants. The preschools selected for this study were targeted due to their high enrolment numbers, their location within the district and their funded by the Department of Social Development. Eight of the largest preschools, four from Klerksdorp and four from Wolmaransstad, were randomly selected in the district. Within the selected preschools, simple random sampling of children was carried out.

### 2.5. Data Collection

#### 2.5.1. Socio-Demographics of Study Participants

The study adapted a questionnaire that was used in nutritional status studies [34,35], which considered the UNICEF conceptual framework for malnutrition [36]. The questionnaire was validated through content and face validity and a pilot study. Independent translators who speak Setswana as their mother tongue and are conversant with English did forward and backward translations of the questionnaire. The questionnaire comprised sociodemographic questions about the personal information of mothers, such as age, marital status, education level, as well as household information of the income, family size, head of household, availability of electricity, and sanitation. In addition, questions focused on obstetric history and children’s characteristics (childbirth date, sex, birth order, and birth weight) and selected feeding practices, mainly on breastfeeding and the introduction of solid foods. Trained research assistants administered questionnaires to mothers in Setswana, a local language in the study area.

#### 2.5.2. Dietary Diversity Score

Dietary diversity score was calculated based on 24 h recall of mothers of the child’s consumption of 12 food groups within the past 24 h (Food and Agriculture Organization, 2007). The food groups included were based on FAO [37] recommendations, as follows: (i) cereals, (ii) vegetables, (iii) fruits, (iv) meat, (v) eggs, (vi) fish and other sea foods, (vii) legumes, nuts and seeds, (viii) milk and milk products, (ix) oil and fats, (x) sweets, (xi) spices, condiments and beverages, and (xii) tubers and roots. Commonly consumed foods in the area were incorporated into each food group. A dietary diversity score was created based on the mother’s recall of the child’s food intake in the previous 24 h. The response option of “yes” was scored one point if at least two food items in each food group were consumed by the child, whereas half a point was awarded for food items consumed once per group. For food groups not consumed at all, with a response option of “no”, zero (0) points were given. Dietary diversity scores (DDS) was summed up by counting each of the 12-food groups, and classified as low (≤4), medium (5–8) and high (9–12). The cut-offs used were due to a lack of national and international guidelines on which to base cut-offs [38].

#### 2.5.3. Anthropometric Measurements and Nutritional Indicators

Anthropometric measurements of height and weight for each child were determined following standard procedures. The weight of children was measured using an infant electronic digital weighing scale (smart D-quip electronic scale) to the nearest 0.1 kg, while standing height was measured to the nearest 0.1 m using a non-stretch tape measure. All measurements were taken three times, and the average recorded. Nutritional status indices were generated through anthropometry conversion to sex specific Z-scores using WHO Anthro Software. Stunting and underweight were defined as low height-for-age (HAZ) and weight-for-age (WAZ), respectively, below −2SD, while normal nutritional status was Z-scores between −2SD to +2SD. BMI-for-age (BAZ) scores between +1SD and +2SD indicated possible risk of overweight. Overweight was at BAZ >+2SD, obesity at BAZ ≥3SD and wasting/thinness was at <−2SD [39].

### 2.6. Statistical Analysis

Statistical analyses were performed using STATA (Intercooled Stata^®^ Version 14). Skewness–Kurtosis tests for normality were performed to check the distribution of data for children (i.e., age, weight, height, HAZ, WAZ and BAZ). Descriptive statistics for weight and height of the children were computed, compared using a parametric *t*-test, and results are presented as mean ± SD. Descriptive statistics for HAZ, WAZ and BAZ of the children were computed and compared by sex using the Mann–Whitney test (i.e., non-parametric test), and the results are presented as median (interquartile range (IQR)). A chi-square test was used to compare the prevalence of stunting, underweight and thinness, stratified by sex, and the results are presented as frequency (*n*) and percentage (%). The descriptive statistics of DDS (i.e., mean ± SD) were compared by sex and age, using independent sample t-test and ANOVA, respectively. The proportions of low and medium DDS were compared by sex and age using a chi-square test. To assess the relationship between dietary diversity and nutritional status indicators (HAZ, WAZ and BAZ, stunting, underweight and thinness), univariate linear regression (for continuous outcomes) and univariate logistic regression (for dichotomous outcomes) models were built. Multivariate models were created, adjusting for the characteristics of children (i.e., sex, age, birth order, term of pregnancy and breastfeeding), maternal (i.e., age, marital status, education level and employment status) and household (family size, monthly income, house type, access to water and toilet type). Models were run for the overall sample of children and in the age subgroups of children (i.e., 3, 4 and 5 years) to assess the influence of dietary diversity on children who may have a higher risk of poor nutritional status. Unadjusted odds ratio (OR) and adjusted odds ratio (AOR) are presented with a 95% confidence interval (CI) and significance was considered at *p* < 0.05.

### 2.7. Ethics Statement

This study was conducted according to the guidelines laid down in the Declaration of Helsinki, and all procedures involving human subjects were approved by Sefako Makgatho Health Sciences University Research and Ethics Committee (SMUREC) (SMUREC/H/47/2019: PG). Furthermore, this study received permission from the North West Provincial Department of Social Development Research Committee, South Africa, and written consent was obtained from the mothers.

## 3. Results

### 3.1. Characteristics of Children and Mothers

Table 1 describes the basic characteristics of the children and mothers. A total of 379 children aged three to five years were included in the analysis. Among the children, 181 (48%) and 198 (52%) were boys and girls, respectively. The mean age of the children was 4 ± 0.7 years. Most of the children were born full term (88%) and 86% were breastfed. Mixed feeding was given to 40% of the children (Table 1). The mean age of mothers was 31 ± 7 years, with 25% aged less than 25 years. Few mothers were employed (28%). Most mothers (80%) were single, unemployed (72%), received a child grant (86%), and had one to four persons in a household (74%). The majority of mothers lived in households with a monthly income of less than USD 296.37 (70%), non-brick houses (64%), with electricity (89%), and access to water (77%) and flush toilets (88%) (Table 1).

### 3.2. Nutritional Status of Children

Mean values for weight and height were not significantly different between boys and girls (*p* = 0.342 and 0.157, respectively. The nutritional status of children was defined by stunting, underweight, and thinness. Overall, the prevalence of stunting, underweight and thinness was 29%, 13% and 6%, respectively. The negative mean Z-scores for height and weight showed a high degree of stunting and underweight in the total population. Comparison of nutritional status by sex revealed that differences in the prevalence of HAZ and WAZ scores were not significantly different, whereas BAZ showed a marginal significance (*p* = 0.057) between boys and girls. Mean values for HAZ (*p* = 0.154) and WAZ (*p* = 0.864) were not significantly different by sex; however, the mean BAZ was significantly different for boys and girls (*p* = 0.002) (Table 2).

### 3.3. Dietary Diversity

#### 3.3.1. Dietary Diversity Scores of the Children by Sex and Age

DDS of children was divided into low (≤4), medium (5–8) and high (9–12). However, due to the small number of preschool children with high DDS (i.e., 1%), the medium and high DDS were combined in the analysis (medium ≥ 5). The DDS ranged from 1 to 10 out of 12 food groups, with a mean DDS of 4.39 ± 1.55. The majority (61%) of children fell within the low DDS group, while 39% were in the medium DDS category (Table 3). The differences in the mean values of DDS according to sex and age were not significantly different. Likewise, comparing the prevalence of DDS of children by sex and age categories showed no significant difference (Table 3).

#### 3.3.2. Diversity by Consumption Frequencies of Food Groups

**Figure 1:**
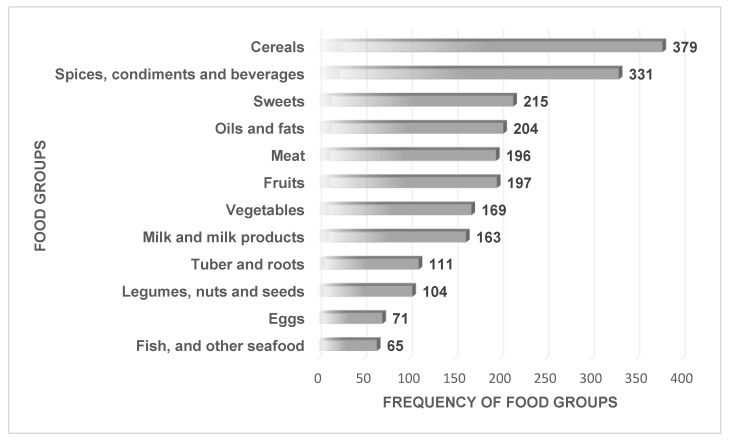
Food groups consumed by children.

The diets of the children were composed mainly of cereals, beverages, sweets, oils and fats, meat and fruits, and these food groups were consumed by more than 50% of the children. The consumption frequencies of the food groups showed that fish and other seafood [*n* = 65 (17%)], eggs [*n* = 71(19%)], and legumes, nuts and seeds [*n* = 104(27%)] were the least consumed, while 100% (*n* = 379) of children consumed a form of cereal and 87% (*n* = 331) consumed beverages (Figure 1).

### 3.4. Association between Dietary Diversity Score and Nutritional Status

Table 4 shows the results of univariate and multivariable models for the association between dietary diversity and nutritional indicators. Overall, no significant association was observed between DDS and the nutritional indicators.

Further stratification of the children by age for the associations between dietary diversity and nutritional indicators is presented in Table 5. In the adjusted model, the association between dietary diversity and nutritional indicators was strong among four-year-old children. A higher dietary diversity score was significantly associated with lower odds of being stunted [AOR = 0.25, 95%CI: 0.10 to 0.92] among four year old children. In the unadjusted model, five-year-old children had significantly lower odds of being underweight [OR = 0.32, 95%CI: 0.57 to 0.07].

## 4. Discussion

The current study investigated the association between dietary diversity score and the nutritional status of preschool children aged three to five years in the North West Province of South Africa, using the FAO scoring system of 12 food groups over a 24 h reference period. The study showed a prevalence of low DDS (69%), mean DDS of 4.39 ± 1.55, and starchy-based staples were the most consumed (100%), and fish and other seafood (17%) were less consumed. The study further reported the prevalence of stunting (29%), underweight (13%) and thinness (6%) among the preschool children. Higher DDS was significantly associated with lower odds of being stunted among the four year olds and WAZ (i.e., underweight) among the five year olds.

The mean DDS (4.39) reported in this study is almost similar to the means reported in other previous studies conducted among the under five children in other developing countries, such as Trinidad and Tobago (4.6) [40], Sri-Lanka (4.56) [41] and Filipino (4.91) [42]. However, these studies used either the 6-food groups or the 9-food groups systems [40,41,42]. Other studies showed higher mean values, using 7-food groups, 9-food groups or 12-food groups [43,44,45]. For instance, a mean DDS of 5.77 has been reported among Chinese children using 9-food groups [45], and a mean DDS of 6.04 using 12-food groups has been reported in Nigeria [44], while a mean DDS of 6.52 was observed among South African children. Furthermore, researchers in South Africa have reported a lower mean DDS (3.6) among children aged one to eight years [25], while a mean DDS of 2.29 using 7-food groups has been reported among children in Ghana [43]. The differences in scoring systems used for the DDS, the types and number of foods, as well as the age of study samples, make a comparison of results across countries difficult. As such, there is a need to apply caution in the interpretation of the DDS.

The prevalence of low DDS among children was 69% in the current study. This finding concurs with previous studies among South Africans in all age groups and settings reported to have low dietary diversity [25,46,47,48,49]. Low dietary diversity is common among under five children from other countries, such as Zambia (62.6%) [50], Madagascar (42.1% and 47.6%) [51], Nigeria (73.5%) [44], and Ghana (47.2%) [43]. Hence, the low dietary diversity reported in the current study could be attributed to poor diets of households composed of a limited number of food groups [5].

Dietary diversity is a good predictor of dietary quality and micronutrient density in children [43,52]. Children in low and middle-income countries are reported to suffer from micronutrient deficiencies, due to poor diet quality [43,52]. The low occurrence of fish and seafood (17%), eggs (19%), legumes (27%), tubers and roots (29%), and milk products (43%) in the diet of the preschool children is a reflection of monotonous and less diverse diets of the households. This is in agreement with data from other countries, showing that children consume predominantly starch-based staples, seasonal fruits, vegetables, and few meat products [10,53,54,55]. A plausible explanation for the inadequate consumption of animal source protein and micronutrient rich foods (vitamins and minerals) in this study could be attributed to economic reasons. The majority of mothers (86%) in the current study depended on a child support grant, and this circumstance creates an inability to purchase nutritious food, which determines households’ food availability, and ultimately affects the feeding practices of children [56]. This could in turn lead to the poor growth and development of the child [57]. Efforts should therefore be made to continue to educate parents and caregivers on the importance of inclusion of animal-source and micronutrient rich foods in the diet of children.

In addition to micronutrient deficiencies, lack of a diversified diet is often associated with poor nutritional and health outcomes in children [18,25,58]. In this population, the prevalence of stunting (29%) and underweight (13%) were observed among children with similar proportions in boys and girls. A comparison of prevalence of DDS within the age groups (three, four, five years) showed similarity in low dietary diversity scores. This could be due to the homogeneity of the poor socioeconomic status common in most of the households that children in the current study live in, as indicated by high unemployment rates (70%), dependence on social grants (86%) and income of less than USD 296.39 (70%). Socio-economical characteristics influence the household food insecurity, and household food access is a key indicator for predicting undernutrition [59]. Therefore, the low dietary diversity observed in all age groups and sex is an indication that these children live in poverty and may not likely meet their nutrient requirements for growth.

Although, overall, no association between DDS and child undernutrition indicators was observed in the current study, in a similar study of Ali et al. [60], stratification of children by age showed significant associations, similar to other studies [58,61]. DDS was significantly associated with the nutritional indicators of stunting and underweight among the four and five year olds, respectively. A higher DDS was significantly associated with lower odds of being stunted among four-year-old children. Stunting begins early in the life of a child and is reflective of longer-term nutritional status [62]. Improving the dietary diversity of children at an early stage in life is important to prevent the chronic malnutrition resulting from stunting. Therefore, the importance of diversifying the diet of children at an early stage in life to prevent chronic malnutrition resulting from stunting cannot be over emphasized. Similarly, lower odds of underweight were observed among four-year-old preschool children with a varied diet. A high DDS was found to be protective against WAZ (underweight) among five year old children, and this is consistent with results of studies among under five children from other countries [58,61]. Underweight in children is mainly caused by inadequate food intake [63,64], poor feeding practices [65], and child rearing practices [66], among other factors, such as repeated infections [67], economic [66], residence [68], social, and cultural factors [69]. It is therefore imperative to improve the diversity of foods consumed by children, in order to reduce various forms of malnutrition.

## 5. Limitations

The study has some strengths and limitations. The cross sectional nature of the study limits the possibility of drawing conclusions based on causal relationships. Again, the study relied on the use of mothers’ recall, which may have introduced recall bias; however, food models and samples of the food were used to help mothers’ recall and reduce this bias. Further, we did not obtain information on the amount/quantity of food consumed. Rather, the dietary diversity score was used as an indicator of the overall quality of the diet of a child, reflecting diversity in the recent diet. The sample population in this present study was limited to one district in the North West Province, thus making it difficult to generalize the findings to all preschool children, considering the fact that South Africa has a cultural and ethnicity diversity, as well as differences in settings. Despite these limitations, this study adds valuable information and provides evidence on the influence of dietary diversity on the nutritional status of preschool children, for which data is lacking in the North West Province of South Africa, particularly in the peri-urban settings.

## 6. Conclusions

The dietary diversity of preschool children was low, and the consumption of common starchy staples in the locality was prevalent. The consumption of animal and plant sources of protein (eggs, fruits, fish and other seafood) and vegetables was inadequate, and this could affect growth and development. Higher dietary diversity was found to be associated with lower odds of being stunted and underweight among preschool children aged four and five years. The findings reinforce the importance of continued nutrition education of mothers, caregivers and preschool staff on the need to ensure the consumption of diverse food sources, in order to improve the nutritional status of children. Further studies are recommended on addressing the association of DDS with the nutritional status, and other factors associated with low dietary diversity among preschool children. Nair et al. [70] have reiterated that the key to success in using dietary diversity as a strategy to tackle hidden hunger is in integrating it with the principles of bioavailability, translated to efficient food synergies, with due emphasis on food accessibility, affordability, and outdoor physical activity/lifestyle modifications.

## Figures and Tables

**Table 1:** Descriptive characteristics of children and their mothers (*n* = 379).

Variables	Category	Frequency	Percentage
Child sex	Girls	181	48
	Boys	198	52
Birth order	First	154	41
	Middle	52	14
	Last	173	46
Full-term baby	Yes	333	88
	No	46	12
Child breast-fed	Yes	326	86
	No	53	14
Length of breast-feeding	Less than 6 months	106	28
	Between 6 and 12 months	75	20
	Above 12 months	144	38
Mixed feeding	Yes	151	40
	No	228	60
Introduction of solid food	Less than 6 months	188	50
	Between 6 and 12 months	169	45
	Immediately at 1 year	22	6
Mothers’ age (years)	<25	93	25
	26–34	168	44
	≥35	118	31
Employment status	Employed	108	28
	Unemployed	271	72
Marital status	Single	303	80
	Married	76	20
Household monthly income	<USD 296.37	267	70
	≥USD 296.37	112	30
Receiving social grant	Yes	326	86
	No	53	14
Level of education	High literacy	350	92
	Low literacy	29	8
Number of household members	1 to 4	281	74
	≥5	98	26
Housing	Brick house	135	36
	Non-brick	244	64
Access to electricity	Yes	338	89
	No	41	11
Access to water	Yes	292	77
	No	87	23
Type of toilet	Flush toilet	333	88
	Pit toilet	46	12

Low literacy (i.e., primary school and did not complete secondary school) and high literacy (i.e., completed secondary school and/or tertiary education).

**Table 2:** Comparison of anthropometric values and nutritional status indicators of children by sex.

Nutritional Status Indicators	All *n* = 379	Boys *n* = 198	Girls *n* = 181	*p*-Value
Weight (kg)	15.2 ± 2.50	15.3±2.60	15.1±2.39	0.342
Height (cm)	98.3 ± 7.04	97.9±7.04	98.9±7.02	0.157
HAZ-mean	−1.29 (−2.14; −0.40)	−1.46 (−2.30; −0.53)	−1.10(1.93; −0.30)	0.154
Normal	258 (68)	127 (64)	131 (71)	0.016
Stunting	109 (29)	66 (33)	43 (24)	
Tallness	12 (3)	5 (3)	7 (4)	
WAZ-mean	−0.72(−1.36; 0.06)	−0.73 (−1.34; 0.07)	−0.71 (−1.45; 0.01)	0.864
Normal	311 (82)	163 (83)	148 (82)	0.796
Underweight	49 (13)	24 (12)	25 (14)	
Growth problem	19 (5)	11 (6)	8 (4)	
BAZ-mean	0.16 (−0.59; 1.07)	−0.34 (−0.44; 1.44)	−0.37 (−0.84; 0.78)	0.002 *
Normal	259 (68)	125(63)	134 (74)	0.057
Thinness	22 (6)	11 (6)	11 (6)	
Overweight risk	70 (19)	42 (21)	28 (15)	
Overweight/obesity	28 (7)	20 (10)	8 (4)	

HAZ = height for age Z-score, WAZ = height for age Z-score, BAZ = height for age Z-score. * indicates a significant difference.

**Table 3:** Dietary diversity of children by sex and age group.

Variables	Sex			
	**All**	**Boys**	**Girls**	***p*-Value**
DDS mean	4.39 ± 1.55	4.4 ± 1.4	4.4 ± 1.7	0.775
Low DDS	230 (61)	121 (61)	109 (60)	0.859
Medium DDS	149 (39)	77 (39)	72 (40)	
**Age Group**				
**Variables**	**3 Years**	**4 Years**	**5 Years**	***p*-Value**
DDS mean	4.6 ± 1.5	4.4 ± 1.6	4.2 ± 1.5	0.533
Low DDS	36 (58)	121 (60)	73 (63)	0.738
Medium DDS	26 (42)	81 (40)	42 (37)	

DDS = Dietary diversity score, Low DDS classified as DDS ≤ 4, Medium DDS classified as DDS ≥ 5.

**Table 4:** Association between dietary diversity and nutritional indicators.

Variable	Univariate ^a^		Multivariate ^b^	
All	OR (95% CI)	*p* Value	AOR (95% CI)	*p* Value
HAZ	−0.05 (−0.18 to 0.07)	0.401	−1.35 (−2.88 to 0.17)	0.081
WAZ	−0.12 (−0.26 to 0.02)	0.087	1.93 (−0.35 to 4.22)	0.097
BAZ	−0.08 (−019 to 0.0.3)	0.146	−1.37 (−2.87 to 0.14)	0.076
Stunting ^**c**^	1.01 (0.64 to 1.60)	0.960	0.61 (0.28 to 1.21)	0.176
Underweight ^**d**^	1.54 (0.85 to 2.84)	0.156	1.95 (0.88 to 5.06)	0.131
Thinness ^**e**^	1.82 (0.78 to 4.36)	0.181	1.15 (0.38 to 3.40)	0.803

**^a^** Estimated with linear regression model for continuous variables or logistic regression model for categorical variables and **^b^** adjusting for the characteristics of children (i.e., sex, age, birth order, term of pregnancy and breastfeeding), maternal (i.e., age, marital status, education level and employment status) and household (family size, monthly income, house type, access to water and toilet type). **^c^** Defined as HAZ <−2 SD, **^d^** defined as WAZ <−2 SD and **^e^** defined as BAZ <−2 SD (HAZ = height for age Z-score, WAZ = height for age Z-score, BAZ = height for age Z-score [39]).

**Table 5:** Univariate and multivariate association between dietary diversity and nutritional indicators stratified by age.

Variable	Univariate ^a^		Multivariate ^b^	
**3-Year-Olds**	**OR (95% CI)**	***p* Value**	**AOR (95% CI)**	***p* Value**
HAZ	0.17 (−0.14 to 0.48)	0.284	−1.57 (−8.45 to 5.31)	0.650
WAZ	−0.06 (−0.37 to 0.25)	0.692	2.52 (−7.58 to 12.62)	0.620
BAZ	−0.16 (−0.40 to 0.08)	0.183	−1.83 (−8.57 to 4.92)	0.590
Stunting ^**c**^	1.38 (0.48 to 4.08)	0.565	1.05 (0.22 to 5.10)	0.948
Underweight ^**d**^	3.01 (0.77 to 11.72)	0.112	2.19 (0.34 to 14.37)	0.411
Thinness ^**e**^	2.80 (0.45 to 17.32)	0.268	1.69 (0.17 to 16.42)	0.649
**4-Year-Olds**	**OR (95% CI)**	***p* Value**	**AOR (95% CI)**	***p* Value**
HAZ	−0.03 (−0.20 to 0.13)	0.706	−1.47 (−3.61 to 0.66)	0.175
WAZ	−0.05 (−0.24 to 0.15)	0.646	2.15 (−1.04 to 5.35)	0.186
BAZ	−0.02 (−0.19 −0.4)	0.765	−1.47 (−3.60–0.67)	0.171
Stunting	0.69 (0.37 to 1.28)	0.238	0.25 (0.10 to 0.92)	0.035 *
Underweight	0.98 (0.41–2.30)	0.954	2.38 (0.65 to 8.72)	0.190
Thinness	0.96 (0.26 to 3.55)	0.948	0.60 (0.12 to 3.02)	0.536
**5-Year-Olds**	**OR (95% CI)**	***p* Value**	**AOR (95% CI)**	***p* Value**
HAZ	−0.23 (−0.46 to 0.02)	0.077	−1.12 (−3.81 to 1.53)	0.398
WAZ	−0.32 (−0.57 to −0.07)	0.013 *	1.38 (−2.70 to 5.46)	0.505
BAZ	−0.17 (−0.37 to 0.04)	0.110	−1.08 (−3.73 to 1.57)	0.420
Stunting	1.83 (0.76 to 4.39)	0.179	1.38 (0.44 to 4.36)	0.585
Underweight	2.04 (0.64 to 6.54)	0.230	1.27 (0.26 to 6.24)	0.771
Thinness	3.20 (0.56 to 18.40)	0.192	2.00 (0.23 to 17.56)	0.530

* indicates a significant association. ^**a**^ Estimated with linear regression model for continuous variables or logistic regression model for categorical variables and ^**b**^ adjusting for the characteristics of children (i.e., sex, age, birth order, term of pregnancy and breastfeeding), maternal (i.e., age, marital status, education level and employment status) and household (family size, monthly income, house type, access to water and toilet type). **^c^** Defined as HAZ<−2 SD, **^d^** defined as WAZ <−2 SD and **^e^** defined as BAZ <−2 SD [39].
